# Objective sleep and cardiometabolic biomarkers: results from the community of mine study

**DOI:** 10.1093/sleepadvances/zpad052

**Published:** 2023-11-28

**Authors:** Steven Zamora, Kelsie M Full, Erica Ambeba, Kimberly Savin, Katie Crist, Loki Natarajan, Dorothy D Sears, Sarah Alismail, Noémie Letellier, Tarik Benmarhnia, Marta M Jankowska

**Affiliations:** Department of Climate, Atmospheric Sciences, and Physical Oceanography, Scripps Institution of Oceanography, UCSD, La Jolla, CA, USA; Division of Epidemiology, Department of Medicine, Vanderbilt University Medical Center, Nashville TN, USA; Herbert Wertheim School of Public Health and Human Longevity Science, UC San Diego, La Jolla, CA, USA; San Diego State University/University of California San Diego Joint Doctoral Program in Clinical Psychology, Department of Psychology, SDSU, San Diego, CA, USA; Urban Studies and Planning Department, San Diego University, San Diego, CA, USA; Herbert Wertheim School of Public Health and Human Longevity Science, UC San Diego, La Jolla, CA, USA; College of Health Solutions, Arizona State University, Phoenix, AZ, USA; Department of Population Sciences, Beckman Research Institute, Duarte, CA, USA; Department of Climate, Atmospheric Sciences, and Physical Oceanography, Scripps Institution of Oceanography, UCSD, La Jolla, CA, USA; Department of Climate, Atmospheric Sciences, and Physical Oceanography, Scripps Institution of Oceanography, UCSD, La Jolla, CA, USA; Department of Population Sciences, Beckman Research Institute, Duarte, CA, USA

**Keywords:** accelerometry, health disparities, quantile regression, metabolic health, cardiovascular health, Latino ethnicity

## Abstract

**Study Objectives:**

Examining multiple dimensions of sleep health may better capture associations between sleep and health risks, including cardiometabolic disease (CMD). Hispanics have elevated risk for inadequate sleep and CMD biomarkers. Few studies have explored whether associations between sleep and CMD differ by Hispanic ethnicity.

**Methods:**

Leveraging data from the Community of Mine (CoM) study, a cross-sectional investigation of 602 ethnically diverse participants, we derived accelerometer-measured sleep duration and efficiency, and self-reported sleep quality. Accelerometer-measured sleep exposures were analyzed both as continuous and categorical variables. Multivariate and quantile regression models were used to assess associations between sleep and CMD biomarkers (insulin resistance, systolic blood pressure, and low-density-lipoprotein cholesterol), controlling for age, sex, ethnicity, education, smoking status, and body mass index. We examined the potential effect modification of Hispanic ethnicity.

**Results:**

We observed mixed results based on CMD biomarkers and sleep exposure. Increased sleep duration was significantly related to low-density lipoprotein cholesterol in adjusted models (estimate = 0.06; 95% CI: 0.02, 0.11). Poor sleep efficiency was associated with greater insulin resistance in the adjusted quantile (estimate = 0.20; 95% CI: 0.04, 0.36) model at the 90th percentile. Self-reported sleep quality was not associated with CMD outcomes. There was no evidence of effect modification by Hispanic ethnicity.

**Conclusions:**

In this cohort, sleep health measures were found to have mixed and at times opposing effects on CMD outcomes. These effects did not demonstrate an interaction with Hispanic ethnicity.

**Statement of Significance**

Sleep is essential for overall health while poor sleep is a risk factor for cardiometabolic disease (CMD); however, few studies use multiple dimensions of sleep. Using objective measurements of sleep in tandem with self-report may shed light on sleep associations with CMD biomarkers. Furthermore, there is a paucity of studies on sleep and CMD outcomes in Hispanic individuals, who are disproportionately more vulnerable to poor sleep and worse CMD outcomes. In this study, we shed light on the complex relationship between multiple dimensions of sleep health and CMD in an ethnically representative cohort, providing insights that can inform interventions to improve CMD health outcomes.

## Introduction

Sleep is an essential physiological function that impacts all aspects of a person’s health. One in three American adults fails to meet the recommended sleep duration guidelines of sleeping 7–9 hours per night [1]. Sleep health is optimally measured using a multidimensional approach that encompasses features such as duration, efficiency, and quality [2]. However, there remains a paucity of research that employs objective metrics to explore the relationship between sleep health and health outcomes including cardiometabolic disease (CMD) [3]. CMD includes a spectrum of conditions such as cardiovascular diseases (CVD), type-2 diabetes, and metabolic disorders. CMD is the number one cause of death worldwide [4, 5].

Short (<7 hours) and long (>9 hours) nightly sleep duration is associated with CMD including elevated biomarker measures of insulin resistance, systolic blood pressure (SBP), and low-density lipoprotein (LDL) cholesterol, as well as increased rates of metabolic syndrome [6–16]. Laboratory-based studies have shown that sleep deprivation is linked with impaired glucose regulation and increasing sleep duration can improve insulin sensitivity [17, 18]. Insufficient sleep may negatively influence blood glucose by inhibiting the circadian rhythm of one’s resting metabolic rate [19]. Research on sleep quality and its association with CMD is less common with inconsistencies in directional associations [20, 21]. Recent studies in a South African population found that objectively measured good sleep efficiency was associated with lower HOMA-IR [22, 23]. These findings may warrant further exploration as a potential intervention opportunity for individuals dealing with insulin resistance.

Sleep can be measured through self-report [2]; however, factors such as mood, perception, and memory can influence the accuracy of reported sleep duration [24–26]. Similarly, self-reported sleep quality can take on a different meaning than actigraphy-measured sleep quality, commonly defined as sleep efficiency. Therefore, the American Academy of Sleep Medicine endorses the use of actigraphy to obtain objective measures of sleep duration and efficiency, and studies have validated their use in comparison to polysomnography (PSG) [27, 28]. The inclusion of both types of measures (self-report and actigraphy) may lead to a more comprehensive picture of how objectively measured and perceived sleep is associated with CMD. Lack of consistent results and measurement modalities in the literature speaks to the need for more studies to investigate multiple dimensions of sleep, and their relationship to biomarkers of CMD.

A second gap in the literature that this study seeks to address is a lack of research on Hispanic ethnic minorities, sleep health, and CMD [29]. Hispanic individuals are disproportionately affected by disparities in sleep duration as well as worse CMD outcomes [30–32]. Among Hispanic adults, lower sleep efficiency, but not duration, has been associated with a greater prevalence of hypertension [13], and shorter sleep duration has been linked to higher levels of hemoglobin A1c [33]. Research suggests that sleep may differentially relate to CMD biomarkers among minoritized racial and ethnic groups [34–37], possibly explaining CMD health disparities. Gaining insight into how sleep relates to CMD in Hispanic populations, and specifically if differences in sleep may be driving ethnic differences in CMD outcomes, may elucidate ways to reduce inequities. The goals of this study are to (1) assess the effects of objectively measured sleep efficiency and sleep duration as well as self-reported sleep quality on CMD-related biomarkers and (2) assess if relationships between sleep measures and CMD biomarkers are modified by Hispanic ethnicity.

## Material and Methods

### Study population

The Community of Mine (CoM) study is an observational, cross-sectional study of 602 participants (40% Hispanic, 56% female, mean age = 59 years) living in San Diego County. Data collection was completed in 2017. The full details and protocol of the study are described elsewhere [38]. Participants wore accelerometer devices for a 2-week period, attended a clinical visit and blood draw, recorded nightly sleep journals, and completed self-report surveys that included demographic information. Hispanic ethnicity was measured with the question “Are you of Hispanic, Mexican, or Latin American descent?” For participants who answered “yes,” the Abbreviated Multidimensional Acculturation Scale was administered which included self-reported culture or origin [39]. Written informed consent was obtained from all participants and ethics of the study were approved by UCSD IRB (protocol #140510).

### Sleep health exposures

Participants were asked to wear an Actigraph GT3X+ accelerometer device on their wrist at all times during the study period. They were asked to complete a sleep journal that included time to sleep and time of waking for each night of the study. Research staff used a standardized protocol to assess valid nights of accelerometer wear time, confirm and/or adjust in-bed and out-of-bed times, and prepare the sleep data for processing (Supplementary Material). The Cole-Kripke algorithm [40] was run to generate nightly sleep variables including sleep duration and efficiency. Total sleep time (TST) over 24 hours was calculated by summing minutes classified as sleep by the Cole-Kripke algorithm. TST was classified as “poor” if nightly sleep was <7 or >9 hours, and “normal” otherwise. Sleep efficiency (i.e. the ease of falling asleep and returning to sleep) was defined as the proportion of the total sleep period classified as sleep. Sleep efficiency was coded as “normal” if it was ≥85% or “poor” if <85%. Valid sleep nights had both accelerometer data and a sleep journal. All accelerometer sleep measures were averaged across valid sleep nights to obtain one set of sleep measures per participant. Self-reported sleep quality was measured using the Patient-Reported Outcomes Information System (PROMIS) sleep questionnaire [41]. It was classified as “normal” for T-standardized scores ≥3 or “poor” if <3. The “normal” classifications of sleep exposures were used as the reference group in statistical analyses.

### CMD biomarkers

Participants fasted for 12 hours prior to a 45 mL blood draw. Samples were kept on ice and centrifuged at 4°C to isolate plasma, serum, and buffy coat from both EDTA and serum tubes. The samples were then aliquoted and stored at −80°C [38]. Insulin resistance was calculated using the Homeostatic Model Assessment of Insulin Resistance (HOMA-IR) according to the formula: fasting plasma insulin (mlU/L) × fasting plasma glucose (mg/dL)/22.5. A full lipid panel was conducted. LDL cholesterol (mg/dL) was used as the CMD biomarker due to its major role in pathogenesis of atherosclerosis [42]. LDL cholesterol refers to the cholesterol composed of LDLs found in blood plasma. Blood pressure was measured three times with a 1-minute interval between readings. A fourth measurement was conducted if two of the three prior readings differed by > 5 mmHg. SBP (mm/Hg) refers to the pressure exerted on the arteries during the contraction, which measures the force exerted by the blood vessels as the heart beats.

### Covariates

Covariates included age, sex (male or female), Hispanic ethnicity (yes or no), education (college degree; some college, vocational school, or high school graduate; or less than high school), current smoking status (yes or no), and BMI (kg/m^2^). BMI categories of normal (≤24.9), overweight (25.0–29.9), and obese (>30.0) were used for descriptive statistics and the continuous BMI variable was used as a covariate in all regression analyses.

### Statistical analysis

Descriptive statistics are presented as either mean and standard deviation (SD) for numerical variables or count and percentage for categorical variables, stratified by ethnicity and for the total sample. Participants were included in analysis if they had all covariate data and accelerometry data with sleep variables. Data were considered missing at random. Independent proportion z-tests assessed differences by Hispanic ethnicity for each binary sleep classification. We used multivariate regression to estimate the associations between sleep exposures (efficiency, duration, and quality) and CMD biomarkers (HOMA-IR, SBP, and LDL cholesterol) in unadjusted and adjusted models (adjusting for age, sex, Hispanic ethnicity, education, current smoking status, and BMI). Accelerometry measured TST and sleep efficiency were analyzed as both continuous and categorical variables (poor vs. normal). HOMA-IR was log-transformed to address the skewness of this variable. An interaction term for self-identified Hispanic ethnicity by sleep exposure was included in the adjusted models to determine if Hispanic ethnicity modified any of the associations. Lastly, quantile regression analyses of the adjusted models were performed to determine whether observed associations differed across quantiles of the outcomes (10th, 25th, 50th, 75th, and 90th). Quantile regression enabled us to extend our analysis by examining the associations between variables at different percentiles of the frequency distribution, including the median [43]. All statistical analyses were performed with SAS statistical software (SAS Institute Inc, Cary, North Carolina), and were two-tailed with a significance threshold set to *p* < 0.05.

## Results

The analytic sample that included all covariate and accelerometer data sleep exposures was 570 participants. Descriptive statistics on these individuals are displayed in Table 1 and are stratified by Hispanic ethnicity (43% of the sample). Of participants who identified as Hispanic, 69% self-reported to be Mexican. Key differences in covariates include higher levels of college education in non-Hispanics compared to Hispanic participants (69.4% vs. 35.8%), lower levels of obesity (28.7% vs. 41.9%), and slightly higher smoking rates (8.3% vs. 6.9%), respectively. Across outcomes, Hispanic participants had higher HOMA-IR values on average compared to non-Hispanic participants (3.9 vs. 2.6) with minor differences observed for LDL cholesterol and SBP. For sleep outcomes, almost 70% of the sample had normal sleep efficiency. However, Hispanic participants had higher rates of poor efficiency (35.4%) compared to non-Hispanic participants (26.5%; *p* = 0.01). Over half of the sample had poor TST (55%) with higher rates of poor sleep observed in Hispanics (59.3%) compared to non-Hispanics (52.5%) (no significant difference). For self-reported sleep quality, Hispanic participants were twice as likely to report poor sleep quality (13.0%) compared to non-Hispanic participants (6.8%; *p* = 0.02).

**Table 1.** Descriptive Statistics (Mean/Standard Deviation or Number/Percent) for the Sample (*n* = 570), Hispanic (*n* = 246), and Non-Hispanic (*n* = 324) Participants. Independent Proportion z-Test Results Comparing Binary Sleep Outcome by Ethnicity

Variable	Total *n* = 570	Hispanic *n* = 246	Non-Hispanic *n* = 324
Age	58.9 (10.9)	56.3 (10.7)	60.9 (10.8)
Sex (female)	316 (55.4%)	150 (61.0%)	166 (51.2%)
Education			
College degree	313 (54.9%)	88 (35.8%)	225 (69.4%)
High school/ vocational	199 (34.9%)	101 (42.7%)	95 (29.0%)
Less than high school	58 (10.3%)	53 (21.5%)	5 (1.5%)
BMI (kg/m^2^)	28.7 (5.9)	29.7 (5.8)	27.9 (5.9)
Normal	161 (28.2%)	52 (21.1%)	109 (33.6%)
Overweight	213 (37.4%)	91 (37.0%)	122 (37.7%)
Obese	196 (34.4%)	103 (41.9%)	93 (28.7%)
Current smoking status	44 (7.7%)	17 (6.9%)	27 (8.3%)
HOMA-IR^+^	3.2 (3.4)	3.9 (3.9)	2.6 (2.9)
LDL Cholesterol (mg/dL)^+^	108.3 (30.8)	109.3 (29.7)	107.5 (31.5)
Systolic blood pressure (mmHg)	127.2 (17.4)	125.6 (17.0)	128.4 (17.5)
Sleep efficiency (objective)	86.7 (6.6)	85.8 (5.9)	87.4 (6.9)
Poor (<85%)	173 (30.4%)	87 (35.4%)^*^	86 (26.5%)
Normal (>85%)	397 (69.6%)	159 (64.6%)	238 (73.5%)
Total sleep time (hours)	6.9 (0.9)	6.9 (0.9)	6.9 (0.9)
Poor (<7 or > 9 hours)	316 (55.4%)	146 (59.3%)	170 (52.5%)
Normal (7–9 hours)	254 (44.6%)	100 (40.7%)	154 (47.5%)
Self-report sleep quality^+^			
Poor	54 (9.6%)	32 (13.0%)^*^	22 (6.8%)
Normal	509 (90.4%)	209 (87.0%)	300 (93.2%)

BMI, body mass index; HOMA-IR, Homeostatic Model Assessment of Insulin Resistance; LDL, low-density lipoprotein.

^*^ *p* < 0.05.

^+^HOMA-IR sample size is 566, LDL cholesterol sample size is 556, and self-report sleep quality sample size is 563.

Table 2 shows multivariate regression results for effects of sleep exposures on CMD biomarkers. Unadjusted multivariate regression models for HOMA-IR showed that participants with poor sleep efficiency, TST, and quality (compared to normal efficiency/TST and good quality) exhibited higher levels of HOMA-IR, but estimates were imprecise. A statistically significant association was found for continuous sleep efficiency (estimate = −0.02; 95% CI: −0.03, −0.01). In adjusted models, this association was attenuated but remained significant. Those with poor sleep efficiency and TST had lower levels of SBP in unadjusted and adjusted models, compared to those with normal efficiency and TST. However, these results lacked precision. When examined as continuous variables, greater efficiency and TST were associated with minor (imprecise) increases in SBP. Self-reported sleep quality showed the opposite relationship, with those reporting poor quality having higher SBP (not significant). Similar findings were observed in the LDL cholesterol models. The continuous measure of TST was significantly related to LDL cholesterol in unadjusted and adjusted models, with increases in sleep associated with increases in LDL cholesterol (estimate = 0.06; 95% CI: 0.02; 0.11).

**Table 2.** Multivariate Regression Results for Effects of Objective Sleep Efficiency (Categorical and Continuous), Objective Total Sleep Time (Categorical and Continuous), and Self-reported Sleep Quality on the Outcomes of HOMA-IR, Systolic Blood Pressure (mm/Hg), and Low-Density Lipoprotein (mg/dL). Models are Presented as Unadjusted and Adjusted for Age, Sex, Education, Body Mass Index, Hispanic Ethnicity, and Smoking Status

Outcome	Sleep exposure	Unadjusted estimate	95% CI	Adjusted estimate	95% CI
HOMA-IR	Efficiency (poor)	0.14	(−0.01 to 0.28)	0.06	(−0.06 to 0.18)
	Efficiency (cont.)	−0.02	(−0.03 to −0.01)	−0.01	(−0.02 to 0.00)
	TST (poor)	0.11	(−0.03 to 0.24)	−0.02	(−0.13 to 0.09)
	TST (cont.)	0.00	(0.00 to 0.00)	0.00	(0.00 to 0.00)
	Quality (poor)	0.03	(−0.20 to 0.26)	−0.01	(−0.20 to 0.17)
SBP	Efficiency (poor)	−3.06	(−6.15 to 0.02)	−2.78	(−5.59 to 0.03)
	Efficiency (cont.)	0.06	(−0.16 to 0.28)	0.08	(−0.11 to 0.28)
	TST (poor)	−1.05	(−3.92 to1.82)	−0.84	(−3.46 to 1.78)
	TST (cont.)	0.02	(−0.01to 0.05)	0.02	(−0.01 to 0.04)
	Quality (poor)	1.13	(−3.74 to 6.00)	0.69	(−3.72 to 5.10)
LDL cholesterol	Efficiency (poor)	−0.13	(−5.42 to 5.67)	−0.88	(−6.43 to 4.67)
	Efficiency (cont.)	0.15	(−0.23 to 0.53)	0.25	(−0.13 to 0.64)
	TST (poor)	−1.83	(−6.96 to 3.30)	−2.20	(−7.36 to 2.96)
	TST (cont.)	0.06	(0.01 to 0.11)	0.06	(0.02 to 0.11)
	Quality (poor)	2.33	(−6.29 to 11.00)	2.54	(−6.06 to 11.14)

TST, Total sleep time.

**Figure 1.**
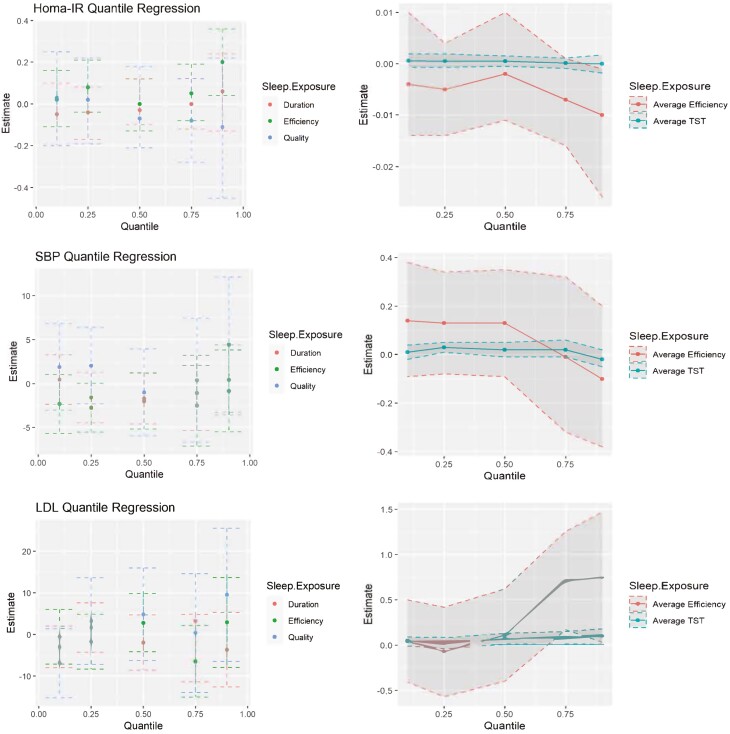
Quantile regression results for effects of poor versus normal sleep efficiency and total sleep time, poor versus good sleep quality (left figures), and continuous sleep efficiency and duration (right figures) on the outcomes of HOMA-IR, systolic blood pressure (mm/Hg), and low-density lipoprotein (mg/dL). Models are adjusted for age, sex, education, body mass index, Hispanic ethnicity, and smoking status.

There was no evidence of significant interaction between any sleep measure and Hispanic ethnicity (Table 3). Estimates for Hispanic ethnicity for Homa-IR were significant for poor sleep efficiency and TST models as well as poor self-reported sleep quality. Additionally, the estimate for poor sleep efficiency as a predictor of SBP became significant in the interaction models. Quantile regression analysis revealed that poor sleep efficiency was significantly associated with HOMA-IR at the 90th quantile (estimate = 0.20; 95% CI: 0.04, 0.36). No other significant associations were observed between categorical sleep exposures and CMD biomarkers in the adjusted quantile regression (Figure 1, Supplementary Table S1). Overall, in Figure 1, we see that continuous TST has precise near-zero effects on all three health outcomes. Stronger associations were observed for the continuous sleep efficiency exposure, but with less precise estimates.

**Table 3.** Multivariate Regression Results (Adjusted for Age, Sex, Education, BMI, and Smoking Status) With Interaction Terms Between Hispanic Ethnicity and Sleep Exposure Effects on the Outcomes of HOMA-IR, Systolic Blood Pressure (mm/Hg), and Low-Density Lipoprotein (mg/dL)

	HOMA-IR		SBP		LDL cholesterol	
Parameter	Adjusted estimate	95% CI	Adjusted estimate	95% CI	Adjusted estimate	95% CI
Efficiency (poor)	0.04	(−0.13 to 0.20)	−4.02	(−7.89 to −0.16)	−0.46	(−8.12 to 7.20)
Hispanic (yes)	0.23	(0.08 to 0.37)	−2.10	(−5.56 to 1.36)	−2.20	(−9.05 to 4.65)
Efficiency (poor) × Hispanic	0.05	(−0.19 to 0.28)	2.61	(−2.98 to 8.21)	−0.89	(−11.95 to 10.17)
Efficiency (cont.)	−0.01	(−0.02 to 0.01)	0.03	(−0.22 to 0.27)	0.22	(−0.26 to 0.71)
Hispanic (yes)	0.52	(−0.96 to 2.01)	−15.28	(−50.61 to 20.05)	−9.08	(−78.27 to 60.10)
Efficiency (cont.) × Hispanic	−0.00	(−0.20 to 0.01)	0.16	(−0.25 to 0.57)	0.08	(−0.72 to 0.88)
TST (poor)	−0.04	(−0.19 to 0.11)	−0.14	(−3.60 to 3.31)	1.43	(−5.37 to 8.23)
Hispanic (yes)	0.22	(0.04 to 0.40)	−0.47	(−4.70 to 3.74)	2.31	(−5.91 to 10.54)
TST (poor) × Hispanic	0.05	(−0.17 to 0.27)	−1.62	(−6.84 to 3.61)	−8.39	(−18.66 to 1.88)
TST (cont.)	0.00	(0.00 to 0.00)	0.02	(−0.02 to 0.05)	0.42	(−0.02 to 0.10)
Hispanic (yes)	0.36	(−0.47 to 1.20)	−3.57	(−23.40 to 16.26)	−24.68	(−63.72 to 14.36)
TST (cont.) × Hispanic	0.00	(0.00 to 0.00)	0.01	(−0.04 to 0.05)	0.54	(−0.04 to 0.15)
Quality (poor)	0.10	(−0.19 to 0.38)	4.29	(−2.45 to 11.03)	3.93	(−9.23 to 17.01)
Hispanic (yes)	0.27	(0.14 to 0.41)	−0.99	(−4.11 to 2.13)	−2.60	(−8.76 to 3.56)
Quality (poor) × Hispanic	−0.19	(−0.56 to 0.19)	−6.24	(−15.10 to 2.61)	−2.41	(−19.70 to 14.87)

For HOMA-IR, there is a downward trend from the 50th quartile onward (Figure 1). Continuous sleep efficiency was related to HOMA-IR (estimate −0.01; 95% CI: −0.03, 0.00) at the 90th quantile (Supplementary Table S1). Similarly, decreasing trends in SBP were observed from the 50th quantile onward. The association between SBP and continuous TST (estimate 0.03; 95% CI: 0.01, 0.05) was significant at the 25th quantile. Unlike HOME-IR and SBP, opposite trend was observed in LDL cholesterol—starting from 50th quartile, as sleep efficiency increases, there is a concurrent increase in LDL cholesterol levels. Significance was observed between continuous efficiency and LDL cholesterol at the 75th (estimate = 0.71; 95% CI: 0.01, 0.13) and 90th (estimate = 0.75; CI: 0.03, 1.47) quantiles. LDL cholesterol was also significantly associated with continuous TST at the 50th (estimate = 0.07; 95% CI: 0.01, 0.13), 75th (estimate = 0.08; 95% CI: 0.01, 0.15), and 90th quantiles (estimate = 0.10; 95% CI: 0.01, 0.18).

## Discussion

Utilizing a combination of self-report and objective measures of sleep health, we examined the association of objectively measured sleep efficiency and TST, and self-reported sleep quality with CMD biomarkers among participants in the CoM study. Results were mixed. We found that better sleep efficiency was associated with decreased HOMA-IR in multivariate regression and quantile regression; however, increased TST was associated with increased LDL cholesterol. We explored interaction effects of Hispanic ethnicity on these associations, which is an important area of investigation given the paucity of sleep research in this population [44]. We found that Hispanics in our sample had objectively measured and self-reported poorer sleep and poorer CMD biomarkers, compared to non-Hispanics. However, sleep differences between Hispanics and non-Hispanics were not significant in explaining CMD biomarkers.

In this study population, we observed statistically significant differences in objectively measured sleep efficiency and self-reported sleep quality between Hispanic participants and non-Hispanic participants. While there were no differences in TST, Hispanic participants had worse accelerometer-measured efficiency and self-reported quality of sleep. Our findings are in line with other research that has observed Hispanic individuals to have poorer sleep health than non-Hispanic individuals, but there is heterogeneity among Hispanic heritage subgroups [44, 45]. Although prior studies have found differences in the associations between sleep and CMD across ethnic groups [31, 32], we did not observe any interactions between Hispanic ethnicity and sleep as related to CMD biomarkers.

The negative association between sleep efficiency and HOMA-IR found in regression analysis was further explored with quantile regression, where we found that the association was most prominent in the 90% quantile of HOMA-IR. This suggests that individuals with already high levels of HOMA-IR may be the most negatively impacted by poor sleep efficiency or may gain the most from increasing sleep efficiency. Previous studies that utilized objective sleep measures have observed a U-shaped association between sleep duration and CMD biomarkers [23, 27]. Inconsistent with these prior findings, we did not observe associations between TST (categorized as poor or normal) and any CMD biomarkers. However, we did find significant associations between CMD biomarkers (LDL cholesterol and SBP) and TST when modeled continuously. Our results are inconsistent with previous studies that have found normal sleep duration and efficiency to be associated with lower SBP [46]. This inconsistency could be attributed to differences in the impact of TST on individuals at different quantiles. We found that longer TST was associated with greater LDL cholesterol in adjusted models and in quantile analyses. The inconsistency in our categorical and continuous analysis results is challenging to interpret. However, our results are consistent with prior studies that have examined associations between sleep exposures and LDL cholesterol, as well as other lipoproteins from serum, and have reported inconsistent or null findings [15, 47, 48]. While self-reported sleep quality was not significant for any measure, the direction and magnitude of associations with SBP and LDL cholesterol were in expected directions. Future research with a larger sample size could explore the precision and accuracy of objective compared to self-reported sleep measures in predicting CMD biomarkers.

This study adds to the growing body of research that utilizes objective sleep measures to investigate associations with biomarkers related to CMD. This study has several strengths including the investigation of sleep efficiency and duration through a multidimensional approach that includes accelerometer-derived metrics and the utilization of a validated measure for assessing self-reported sleep quality in an ethnically diverse sample of adults. Additionally, we incorporated gold-standard visual inspection and coding of nighttime accelerometer data. However, this study is not without limitations. It is cross-sectional in nature and the small sample size may limit the generalizability of our results. Potential confounding variables such as the use of blood pressure medication and statins for cholesterol, were not adjusted for due to data limitations. Although the use of actigraphy to monitor sleep is highly correlated with polysomnography [49, 50], actigraphy is less accurate than polysomnography. Furthermore, it is worth noting that the optimal sleep duration leading to better health outcomes may vary for everyone.

## Conclusion

The mechanisms underlying the relationship between sleep exposures and CMD are not fully understood. This paper extends previous literature by including multidimensional exposures of sleep health with both accelerometer and self-reported measures of sleep, and CMD biomarkers in a diverse sample. We did not find consistent associations between sleep health measures and CMD biomarkers, and associations varied across quantiles of CMD biomarkers, particularly for HOMA-IR and LDL cholesterol. Though no significant effect modification was detected based on Hispanic ethnicity, future studies should continue to investigate racial/ethnic disparities in sleep health and CMD, as these populations are disproportionately affected by inadequate sleep and negative CMD health outcomes.

## Supplementary Material

zpad052_suppl_Supplementary_Tables_S1
