# Supplementary material for: Objective sleep and cardiometabolic biomarkers: results from the community of mine study
Source: Sleep Adv. 2023 Nov 28;4(1):zpad052. doi: 10.1093/sleepadvances/zpad052 (PMC10721447; doi:10.1093/sleepadvances/zpad052)
Supplement: zpad052_suppl_Supplementary_Tables_S1 [file zpad052_suppl_supplementary_tables_s1.docx]

**Objective Sleep and Cardiometabolic Biomarkers: Results from the Community of Mine Study**

Steven Zamora^1^, Kelsie M. Full^2^, Erica Ambeba^3^, Kimberly Savin^5^, Katie Crist^4^, Loki Natarajan^3^, Dorothy D. Sears^6^, Sarah Alismail^7^, Noémie Letellier^1^, Tarik Benmarhnia^1^, Marta M. Jankowska^7^*

^1^ Scripps Institution of Oceanography, UCSD, 9500 Gilman Drive, La Jolla, CA

^2^ Division of Epidemiology, Department of Medicine, Vanderbilt University Medical Center, 2525 West End Ave. Nashville TN 37203

^3^ Herbert Wertheim School of Public Health and Human Longevity Science, UC San Diego, La Jolla, CA, 92093

^4^ San Diego University, Urban Studies and Planning Department, San Diego, CA

^5^San Diego State University/University of California San Diego Joint Doctoral Program in Clinical Psychology, San Diego, CA

^6^ College of Health Solutions, Arizona State University, Phoenix, AZ

^7^ Population Sciences, Beckman Research Institute, City of Hope, 1500 E Duarte Rd, Duarte, CA 91010

*Corresponding Author

Marta M. Jankowska

Population Sciences

Beckman Research Institute of City of Hope,

1500 E Duarte Rd, Duarte, CA 9101

mjankowska@coh.org

**Supplementary Materials**

**Wrist-worn accelerometry sleep data processing**

Processing and scoring of 24-hour wrist-worn accelerometer data collected during the Community of Mine Study was completed by trained staff. Study participants wore the accelerometer device on their wrists continuously for two separate 7-day periods for 24 hours a day, resulting in raw GT3x data files, which were then converted to 60-second AGD files with set parameters. Once processed, data were used to create various sleep variables using the Actilife program including sleep duration (TST), sleep latency, sleep efficiency, sleep fragmentation, and wake after sleep onset (WASO).

The primary objectives of the protocol were:

1. Define major sleep periods: Rectification of self-reported “time in bed” and “time out of bed” from sleep journals with accelerometer data.
2. Running the Cole-Kripke algorithm to determine minute-by-minute sleep/wake time and sleep variables.
3. Create sleep score reports with the following variables: sleep duration (TST), sleep latency, sleep efficiency, sleep fragmentation, wake after sleep onset (WASO).

The accelerometer data in ActiLife was presented in graphs, indicating activity counts, light exposure, and scored sleep periods. The graphs display activity counts on a scale of 0-4000 for 24 or 48 hours of wear. Since the 24-hour window wasn’t exclusively related to nighttime sleep, sleep was divided between two graphs. Activity was measured at one-minute intervals (recorded in 60-second epochs), with blue bars representing activity counts and yellow bars indicating light exposure. The sleep period was highlighted in coral and visually marked as "scored" in green.

Sleep data involves comprehending sleep journals and confirming the alignment of journal entries with accelerometer data for each night of a 7-day wear period (6 nights of sleep). Participants completed sleep journals that included time in bed and time out of bed. Careful analysis was completed to identify patterns and discrepancies in participant entries, as some may lack a consistent sleep schedule or report late bedtimes. To ensure accurate sleep period definition a cross-check of the night of the week with the corresponding data was performed. In some cases, participants may have mistakenly reported the wrong night for their sleep journal, resulting in discrepancies between the journal entries and data. These discrepancies, such as consistent day-to-day matches or shifts in in-bed and out-of-bed times were noted in the comments box for further review and rectified by agreement between two study staff members.

Next the accelerometer AGD files were opened in ActiLife's Sleep Tab and processed using the Cole Kripke algorithm for sleep-wake time scoring and adjusting the activity scale. Participant's sleep journal entries were verified to match the accelerometer data dates, and sleep periods based on the sleep journal's defined sleep periods were entered. Each sleep period was visually reviewed ensuring alignment with the accelerometer data. Non-wear nights were identified and removed. After defining sleep periods and annotating data, a sleep report was generated, providing a detailed analysis of sleep patterns across all nights with accelerometer wear data and corresponding sleep journals.

**Supplement Table 1.** Quantile regression analysis for effects of objective sleep efficiency (categorical and continuous), objective total sleep time (categorical and continuous), and self-reported sleep quality on the outcomes of HOMA-IR, systolic blood pressure (mm/Hg), and low-density lipoprotein (mg/dl). Models are adjusted for age, sex, education, body mass index, Hispanic ethnicity, and smoking status.

|  |  | **HOMA-IR** | | **SBP** | | **LDL Cholesterol** | |
| --- | --- | --- | --- | --- | --- | --- | --- |
| **Sleep Exposure** | **Quantile** | **Estimate** | **95% CI** | **Estimate** | **95% CI** | **Estimate** | **95% CI** |
| Efficiency (poor) | 0.1 | 0.03 | (-0.11 – 0.16) | -2.33 | (-5.70 – 1.02) | -0.53 | (-7.12 – 6.04) |
|  | 0.25 | 0.08 | (-0.04 – 0.21) | -2.76 | (-5.54 – 0.03) | -1.73 | (-8.37 – 4.91) |
|  | 0.5 | 0.00 | (-0.13 – 0.12) | -1.99 | (-5.17 – 1.18) | 2.82 | (-4.17 – 9.80) |
|  | 0.75 | 0.05 | (-0.08 – 0.19) | -2.52 | (-7.10 – 2.05) | -6.48 | (-15.0 – 2.09) |
|  | 0.9 | 0.20 | (0.04 – 0.36) | -0.85 | (-5.52 – 3.83) | 2.90 | (-7.93 – 13.7) |
| Efficiency (cont.) | 0.1 | 0.00 | (-0.01 – 0.01) | 0.14 | (-0.09 – 0.38) | 0.04 | (-0.41– 0.50) |
|  | 0.25 | -0.01 | (-0.01 – 0.00) | 0.13 | (-0.08 – 0.34) | -0.07 | (-0.57 – 0.42) |
|  | 0.5 | 0.00 | (-0.01 – 0.01) | 0.13 | (-0.09 – 0.35) | 0.11 | (-0.40 – 0.62) |
|  | 0.75 | -0.01 | (-0.02 – 0.00) | -0.01 | (-0.32 – 0.32) | 0.71 | (0.17 – 1.26) |
|  | 0.9 | -0.01 | (-0.03 – 0.00) | -0.10 | (-0.38 – 0.20) | 0.75 | (0.03 – 1.47) |
| Total sleep Time (poor) | 0.1 | -0.05 | (-0.20 – 0.10) | 0.44 | (-2.38 – 3.27) | -3.02 | (-8.02 – 1.98) |
|  | 0.25 | -0.04 | (-0.17 – 0.08) | -1.60 | (-4.47 – 1.26) | 1.64 | (-4.32 – 7.61) |
|  | 0.5 | 0.00 | (-0.12 – 0.12) | -1.69 | (-4.61– 1.23) | -1.94 | (-8.58 – 4.69) |
|  | 0.75 | 0.00 | (-0.12 – 0.12) | -1.06 | (-5.35 – 3.23) | 3.30 | (-11.4 – 4.82) |
|  | 0.9 | 0.06 | (-0.13 – 0.24) | 0.41 | (-3.57 – 4.39) | -3.68 | (-12.6 – 5.28) |
| Total sleep time (cont.) | 0.1 | 0.00 | (0.00 – 0.00) | 0.01 | (-0.02 – 0.04) | 0.05 | (-0.01 – 0.09) |
|  | 0.25 | 0.00 | (0.00 – 0.00) | 0.03 | (0.01 – 0.05) | 0.02 | (-0.04 – 0.08) |
|  | 0.5 | 0.00 | (0.00 – 0.00) | 0.02 | (-0.01 – 0.05) | 0.07 | (0.01 – 0.13) |
|  | 0.75 | 0.00 | (0.00 – 0.00) | 0.02 | (-0.01 – 0.06) | 0.08 | (0.01 – 0.15) |
|  | 0.9 | 0.00 | (0.00 – 0.02) | -0.02 | (-0.05 – 0.02) | 0.10 | (0.01 – 0.18) |
| Quality (poor) | 0.1 | 0.03 | (-0.20 – 0.25) | 1.90 | (-3.02 – 6.82) | -6.83 | (-15.1 – 1.41) |
|  | 0.25 | 0.02 | (-0.19 – 0.22) | 2.03 | (-2.32 – 6.38) | 3.21 | (-7.17 – 13.60) |
|  | 0.5 | -0.02 | (-0.21 – 0.18) | -1.01 | (-5.94 – 3.92) | 4.84 | (-6.30 – 15.98) |
|  | 0.75 | -0.08 | (-0.28 – 0.12) | 0.38 | (-6.68 – 7.43) | 0.37 | (-13.88 – 14.61) |
|  | 0.9 | -0.11 | (-0.45 – 0.22) | 4.42 | (-3.28 – 12.13) | 9.52 | (-6.50 – 25.54) |
